# Carbon Nanofiber Growth Rates on NiCu Catalysts: Quantitative Coupling of Macroscopic and Nanoscale In Situ Studies

**DOI:** 10.1021/acs.jpcc.3c02657

**Published:** 2023-08-04

**Authors:** Tom A. J. Welling, Suzan E. Schoemaker, Krijn P. de Jong, Petra E. de Jongh

**Affiliations:** Materials Chemistry & Catalysis, Debye Institute for Nanomaterials Science, Utrecht University, Universiteitsweg 99, 3584 CG Utrecht, The Netherlands

## Abstract

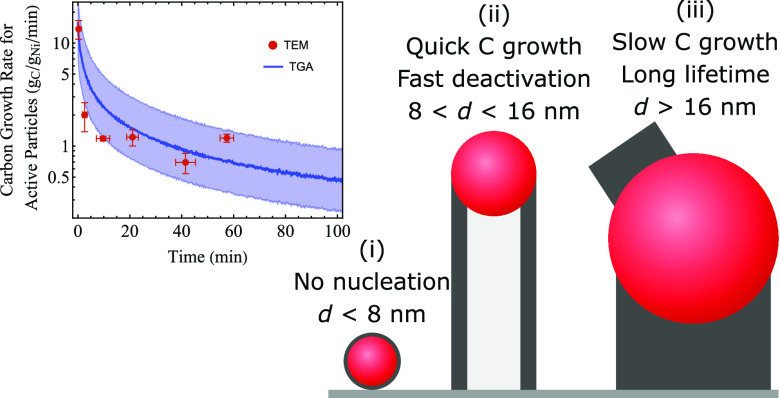

Since recently, gas-cell transmission electron microscopy allows for direct, nanoscale imaging of catalysts during reaction. However, often systems are too perturbed by the imaging conditions to be relevant for real-life catalyzed conversions. We followed carbon nanofiber growth from NiCu-catalyzed methane decomposition under working conditions (550 °C, 1 bar of 5% H_2_, 45% CH_4_, and 50% Ar), directly comparing the time-resolved overall carbon growth rates in a reactor (measured gravimetrically) and nanometer-scale carbon growth observations (by electron microscopy). Good quantitative agreement in time-dependent growth rates allowed for validation of the electron microscopy measurements and detailed insight into the contribution of individual catalyst nanoparticles in these inherently heterogeneous catalysts to the overall carbon growth. The smallest particles did not contribute significantly to carbon growth, while larger particles (8–16 nm) exhibited high carbon growth rates but deactivated quickly. Even larger particles grew carbon slowly without significant deactivation. This methodology paves the way to understanding macroscopic rates of catalyzed reactions based on nanoscale in situ observations.

## Introduction

The consequences of climate change and the potential of hydrogen as an energy carrier instigated interest in CO_*x*_-free hydrogen generation.^1−5^ A promising pathway is the catalytic decomposition of methane over supported metal nanoparticles (NPs) such as Ni, Co, and Fe and their alloys. This process has the potential to produce useful carbon nanomaterials next to hydrogen gas. These carbon nanofibers (CNFs) have great properties such as their strength and conductivity.^1,4^ They have potential as an additive to polymers,^6,7^ in materials for energy and gas storage,^8^ water treatment,^9^ supercapacitors,^10^ and as catalyst support.^11^

A considerable amount of research has been published on methane decomposition.^1,4^ Typically, characterization techniques such as X-ray diffraction, temperature-programmed reduction, and transmission electron microscopy (TEM) have been used to characterize the catalysts (and products). Catalytic tests have been conducted to determine the activity and deactivation of the catalyst.^12^ Chen et al. found an optimal average particle size of ∼34 nm under given conditions,^13^ in line with Takenaka and co-workers.^14^ Additionally, it was found that bimetallic NiCu catalysts have improved lifetimes compared to Ni catalysts.^15−17^ The addition of Cu favors carbon diffusion through the particle^16^ and prevents encapsulation of the particles with carbon.^15^ In our previous work, an optimal carbon yield was found for NiCu catalysts under reaction conditions at the crossover point between two temperature regimes.^17^ Samples are typically heterogeneous in particle size and composition, and studies of the activity of single particles within a catalyst are rare. To improve our understanding of the overall catalyst performance, we need to understand what happens at the individual NiCu nanoparticle level within the catalyst during the reaction.

Recently, the option to observe the catalyst during reaction in real space and real time under realistic conditions has become available due to the emergence of gas-cell electron microscopy.^18−22^ A gas cell consists of strong, electron-transparent windows that can keep the gaseous environment separated from the high vacuum of the electron microscope. In situ TEM studies on the metal nanoparticle-catalyzed growth of carbon nanofibers have been conducted.^23−34^ These studies usually focused on single particles growing fibers and yielded insights such as the active phase of the nanoparticles, or the growth mechanisms of fibers.^31−34^ For example, Huang and co-workers showed oscillatory growth behavior for carbon nanotubes growing from nanoparticles with Fe_3_C as the active phase.^31^ Lyu et al. identified Ni_3_C as the active phase of their nanoparticles that grew fibers, using electron microscopy imaging, diffraction, and electron energy loss spectroscopy.^34^ Fan and co-workers related the dynamic state of Ni–Co particles to the structure of the fishbone fibers grown.^33^ However, individual particle (size) effect studies and a direct comparison to a reaction on a relevant scale in realistic conditions have so far been lacking.

In this work, we performed in situ TEM experiments where we followed more than 60 NPs that were growing CNFs. We compared the nanoscale observations of carbon-supported NiCu particles during the reaction in a gas-cell nanoreactor to in situ thermogravimetric analysis (TGA) experiments on a milligram scale outside the microscope quantitatively. Studying the behavior of ensembles of individual metal catalyst particles at the nanoscale allowed us to understand the overall growth rates, as observed in the macroscopic TGA reactor.

## Methods

### Catalyst Preparation

The catalysts were prepared via incipient wetness impregnation. The metal precursor solutions were obtained by dissolving Ni(NO_3_)_2_·6H_2_O or Cu(NO_3_)_2_·3H_2_O in 0.1 M HNO_3_. To prepare the bimetallic catalyst, a mixture of 3:1 Ni/Cu molar ratio was made. A carbon support, GNP-500, a high surface area (500 m^2^ g^–1^) graphite powder, was impregnated with the precursor solution. After impregnation, the sample was dried under a dynamic vacuum overnight. Thereafter, the sample was heat treated at 330 °C for 2 h under nitrogen flow (200 mL min^–1^ g_cat_^–1^) and reduced at 280 °C for 3 h in 5% H_2_/N_2_ (200 mL min^–1^ g_cat_^–1^).

### Thermogravimetric Analysis Experiments

The methane decomposition experiments were performed with a thermogravimetric analyzer (TGA) (PerkinElmer TGA 8000). Approximately 1 mg of catalyst was loaded in a ceramic crucible samples holder (*V* = 22 μL). The sample weight was monitored during the reaction. First, the catalyst was dried at 70 °C for 15 min under argon, after which the sample was heated to 300 °C (5 °C min^–1^). The sample was reduced at 300 °C for 1.5 h in 5% H_2_/Ar (*F*_total_ = 167 mL min^–1^). Then, the sample was heated to the reaction temperature (5 °C min^–1^) under Ar. As soon as the reaction temperature was reached, methane was introduced to the system by an external gas mixing device. This allowed us to use the same gas composition of 5% H_2_, 45% CH_4_, and 50% Ar at 1 bar as in the in situ TEM experiments. The experiments were carried out with a total flow rate of 167 mL min^–1^ at a total pressure of 1 bar.

### Gas-Cell Electron Microscopy Experiments

The gas-cell TEM experiments were performed in a dedicated gas-cell system (Protochips Atmosphere 210) including a sample holder, gas supply system, and a heating control unit. The cell within the sample holder consists of a top and bottom chip, which were joined using o-rings to separate the airtight inner cell compartment from the high vacuum of the microscope. One of the chips contains a silicon carbide-based heating membrane used for closed-loop temperature control by using the resistance of the silicon carbide. Both chips contain 30–50 nm thick silicon nitride windows, which allows for imaging with the electron beam while containing the gas within the cell. The gas supply system has tanks in which gases can be mixed before they are flowed toward the sample holder.

The experiments were performed on a Talos F200X instrument (Thermo Fisher Scientific), equipped with a field-emission gun operated in TEM mode at 200 kV. The dose rate was 8 e^–^ Å^–2^ s^–1^ unless otherwise specified. A Ceta 4k by 4k camera was used to acquire bright-field TEM images, with an exposure time of 2 s. The images were acquired with TEM imaging and analysis software (TIA) with 2048 × 2048 pixels.

The two chips were glow-discharged (Cressington Power Unit 208) for 30 s prior to the experiment. Then, a dilute prereduced catalyst in ethanol was drop-cast ∼20 times (0.5 μL per time) on the chip containing the heating membrane. We waited slightly between drops in order for the previous drop to dry. In this way, some catalyst flakes were deposited on the electron-transparent windows. Subsequently, the cell was assembled in a dedicated holder and checked for potential leaks.

After the holder was inserted into the microscope, it was first flushed with Ar for 5 min at 0.1 sccm at 1 bar. Subsequently, an in situ reduction step in 5% H_2_ and 95% Ar at 1 bar (flowed at 0.5 sccm) at 300 °C was performed for 75 min. The temperature was then increased to the reaction temperature (550 °C) of 2 °C/s. The gas was subsequently switched to 5% H_2_, 45% CH_4_, 50% Ar at 1 bar flowed at 0.5 sccm. The reaction conditions were typically held constant for over an hour to follow the reaction in situ.

### Microscopy Data Analysis

The data analysis was performed in ImageJ (version 1.53c). In order to extract the average growth rate of CNFs growing from individual particles in g_C_ g_Ni_^–1^ min^–1^, we measured: (i) the amount of Ni in a particle, (ii) the fiber length, (iii) the inner and outer fiber diameter, (iv) the time duration of the growth, and (v) the time after the start of the reaction when the growth occurred. The amount of Ni in a particle was calculated from the diameter of the particle, assuming the particle was spherical. This was often the case in at least some of the frames. It was observed that no large deviations in Ni/Cu ratio existed between particles (Figure S7). This meant the diameter was enough to obtain roughly the amount of Ni per particle without the need for energy-dispersive X-ray spectroscopy (EDX) analysis on every single particle.

To measure the CNF length, we identified a reference feature on a CNF in the first frame. We measured the length from this reference feature to the particle growing the CNF. We then measured the distance between the reference feature and the particle a few minutes later. If the particle deactivated in less than a few minutes, then only the time before deactivation was used to determine the growth rate. The change in length divided by the time between the first and second frames was used to calculate the average CNF growth rate for that particle. The particles often changed growth direction within this time frame, which led to CNFs that are not straight. In those cases, the distance from the reference feature to the first corner was measured, followed by the distances between corners, and last the distance between the last corner and the particle. This is possible with the segmented-line feature in ImageJ. Sometimes multiple carbon structures grew from the same particle at the same time. The sum of the changes in length of all of these structures was then taken to determine the carbon structure length growth rate from that specific metal particle.

The inner and outer fiber diameters were determined by a “plot profile” (15 pixels thick, ∼10 to 15 nm) over the width of the fiber. The inflection points in the gray value profile were identified to determine the inner and outer fiber diameter. The position of the “plot profile” was chosen to best represent the width of the fiber during the whole growth period for which the CNF length was measured. To estimate the error in the determination of the growth rate in g_C_ g_Ni_^–1^ min^–1^, the procedure was repeated 5 times for several particles. We found that the error of measurement was less than 5%.

## Results and Discussion

### Catalyst Characterization

**Figure 1:**
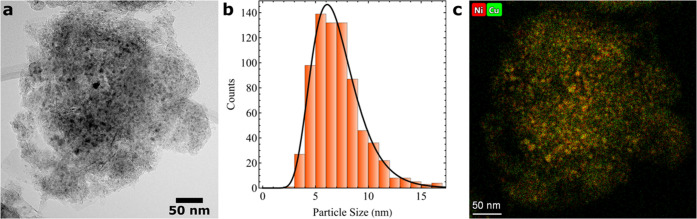
(a) Bright-field TEM image of the reduced catalyst containing NiCu (11 wt % Ni, 4 wt % Cu) particles on a carbon support. (b) Size distribution of the reduced catalyst. The volume-averaged particle size was 7.9 ± 2.9 nm. (c) EDX map of the net distribution of Ni and Cu species over the catalyst.

Figure 1 shows a typical part of the catalyst used in this work. It was prepared via incipient wetness impregnation and subsequently vacuum-dried, heat treated, and reduced. The TEM image in Figure 1a shows that NiCu particles were well spread out over the carbon support (GNP-500). It is an inert support, and the low contrast of the carbon is also ideal for imaging particles on the support in in situ TEM experiments. The particles had a volume-averaged diameter of 7.9 ± 2.9 nm after reduction (Figure 1b). The catalyst contained 11 wt % Ni and 4 wt % Cu and the individual particles were bimetallic NiCu particles, both before and after the reaction (Figures 1c and S6). This ratio was chosen as previous studies have indicated that this is close to the optimal ratio for a high carbon yield.^15,35,36^ Our previous work also showed that this bimetallic NiCu catalyst performed better than a monometallic Ni catalyst.^17^ Detailed characterization of this particular batch of catalyst, including ICP analysis to determine the macroscopic Ni and Cu content, can also be found in our previous work.^17^

### In Situ Gravimetric Experiments

**Figure 2:**
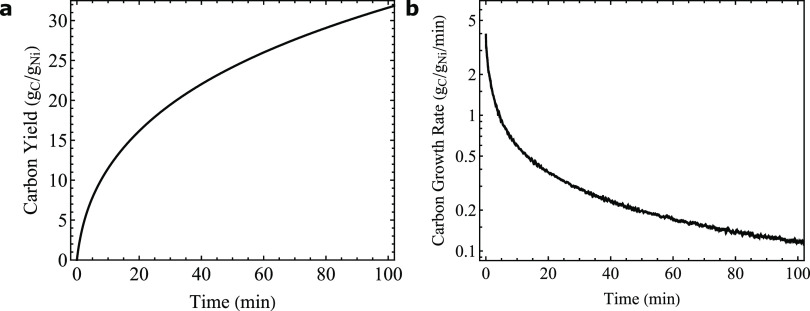
Thermogravimetric analysis at 550 °C in a feed containing 5% H_2_, 45% CH_4_, and 50% Ar at 1 bar. The flow rate was 167 sccm, resulting in GHSV ≈10^10^ h^–1^. (a) Carbon yield. (b) Carbon growth rate versus time.

To obtain statistically relevant information, we performed in situ TGA experiments. We loaded ∼1 mg of sample, which translates to ∼10^12^ NiCu NPs. Figure 2 shows the carbon yield on carbon-supported NiCu catalysts at 550 °C measured via in situ TGA. The feed contained 5% H_2_, 45% CH_4_, and 50% Ar at 1 bar. Argon was used in the feed as a balance. Hydrogen was added to allow direct comparison with the in situ TEM measurements, which require some hydrogen in the feed (Figure S1). The carbon yield increases relatively quickly at the start of the reaction, while carbon grows more slowly at later stages. Figure 2b shows that the carbon growth rate, reported in g_C_ g_Ni_^–1^ min^–1^, decreased to 50, 25, 10, and 5% of the initial rate within 1, 4, 18, and 50 min, respectively. This indicates that the growth rate decreases quickly in the first few minutes, but much slower later in the reaction. Such a fast deactivation at the start of the reaction, but a slower steady growth rate has been described before in the literature.^37−39^ For example, Cazaña et al. found similar curves for a NiCu catalyst on carbonaceous supports in various conditions, although their carbon growth rate does not remain as high as in our case. In the next section, we set out to directly compare the evolution of the carbon growth rate measured from TGA to observations during the reaction with in situ TEM, which helps to understand the underlying phenomena.

### In Situ TEM Observations

#### Establishing Reliable Imaging Conditions

First, the conditions for which we were able to image the catalyst had to be investigated, as the electron beam has the potential to influence results drastically, especially for in situ experiments.^21,40−42^Figure S1 shows several in situ TEM experiments at 600 °C at 1 bar for various gas feed compositions. When hydrogen was not present, CNF growth was observed only in areas that were not irradiated by the electron beam. However, for feeds containing hydrogen (next to methane and argon), carbon nanofiber growth occurred in both imaged and nonimaged areas. We found that a ratio of 1:9 of H_2_ to CH_4_, which is more representative of industrially relevant conditions^12^ than many previous in situ TEM studies, was enough to accurately image CNF growth. As such, we were able to perform experiments with a supported catalyst at atmospheric pressure and a low percentage of hydrogen gas, which was key to compare our experiments to larger-scale experiments outside the microscope.

After H_2_ gas was established as a crucial component to enable imaging of CNF growth, the electron dose rate was optimized. The influence of the electron beam on the growth rate of fibers in these conditions (at 550 °C in 5% H_2_, 45% CH_4_, 50% Ar at 1 bar) had to be investigated.^21^ The results, which are derived from Movie S1, are shown in Figure S2. At extremely low electron dose rates (<1 e^–^ Å^–2^ s^–1^) the carbon structure length growth rate is constant at ∼31 nm/min. However, for higher electron dose rates (>10 e^–^ Å^–2^ s^–1^) the carbon structure growth length increases with the electron dose rate, clearly showing an influence of the electron beam on the carbon growth. Figure S2 shows that it is indeed an electron beam effect rather than an influence of the time that had passed since the start of the reaction or a slight deviation in the average particle size measured. Interestingly, the electron dose rates for which the carbon growth was accelerated by the electron beam were still 1 or 2 orders of magnitude lower than in earlier studies, which is explained by the fact that these studies involved high-resolution imaging, and thus required higher electron dose rates.^23,31,33,43^ The increased growth rate was likely caused by radiolysis of the gas phase, which makes the gas more reactive.^21,22,44−46^ This hypothesis is supported by the drastic changes in electron beam effects upon the addition of hydrogen to the feed. This has implications for studies performed at high electron dose rates. For instance, the dissociation of the first hydrogen from methane is often considered the rate-limiting step.^3,47−49^ If the radiolysis caused by the electron beam already results in a methyl radical and a hydrogen atom from methane in the gas phase rather than only when adsorbed on a catalyst, then this rate-limiting step is circumvented. This is why we took extreme care while choosing the conditions and compared our gas-cell TEM experiments to in situ TGA experiments. The carbon growth rate was found to be unaffected by the electron beam when the electron dose rate was below 10 e^–^ Å^–2^ s^–1^, which we therefore deemed as “safe” in order to image the reaction. As such, our experiments were performed with electron dose rates just below this limit for sufficient resolution without the electron beam influencing the carbon growth rate.

#### Typical Experiment

**Figure 3:**
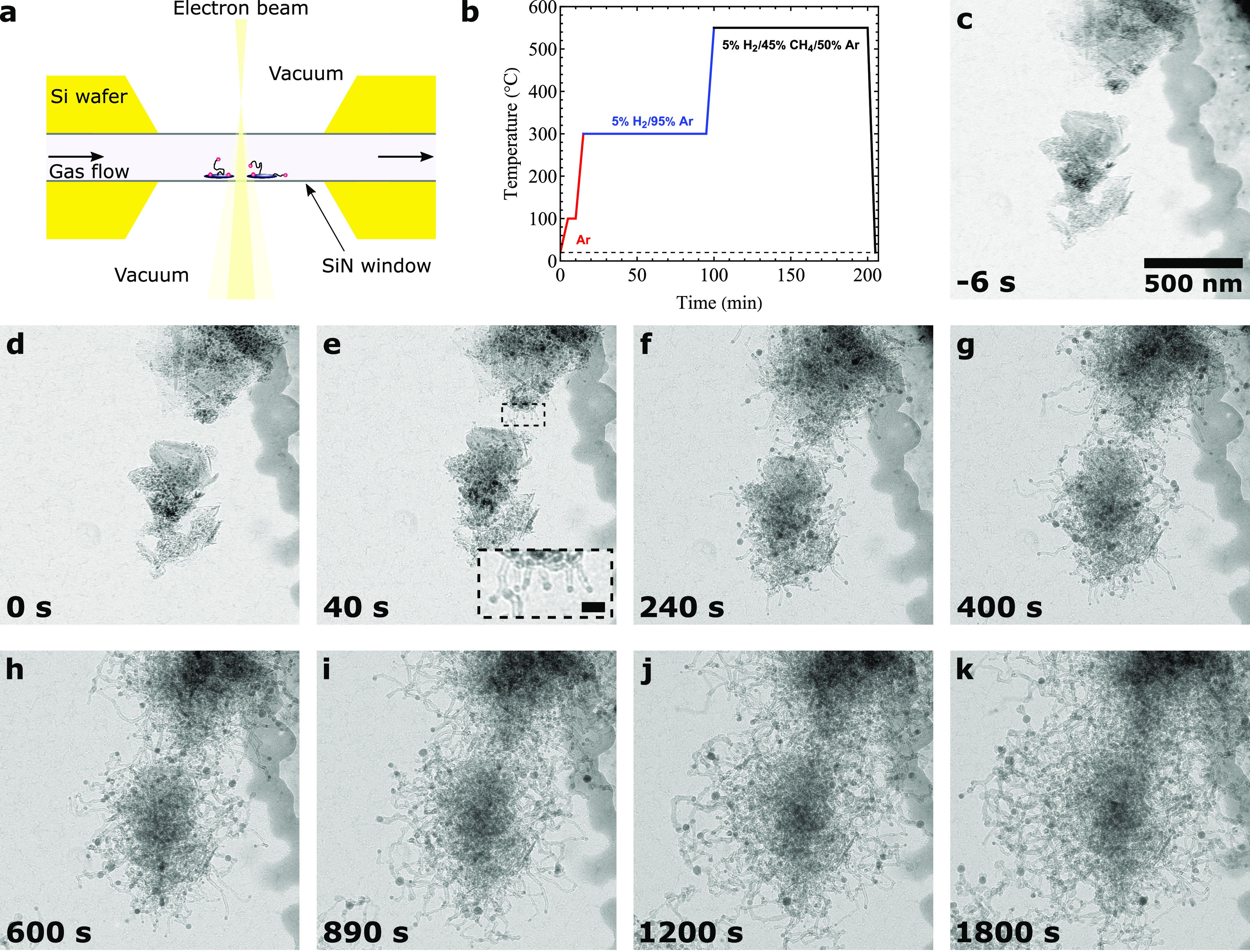
(a) Illustration of the gas-cell TEM nanoreactor. (b) Temperature profile and various gases used during a typical experiment. (c–k) Frames taken at 550 °C with a feed of 5% H_2_, 45% CH_4_, and 50% Ar at 1 bar. The electron dose rate was 7.7 e^–^ Å^–2^ s^–1^. The reaction gas was introduced at *t* = 0 s. The scale bar in the inset of the panel at 40 s is 20 nm.

The gas-cell TEM experiments were performed at 550 °C in 5% H_2_, 45% CH_4_, and 50% Ar at 1 bar to compare directly to the TGA experiments. A schematic of the gas cell is shown in Figure 3a. Figure 3b shows the typical temperature profile of an experiment within the microscope, including which gases are present. First, the sample was dried under Argon. Next, an additional in situ reduction was performed for typically 75 min, after which the temperature was raised to the reaction temperature. When the reaction temperature had been reached, the reaction gas was introduced at *t* = 0 s. Frames of a typical experiment as a function of time are shown in Figure 3c–k, which are analyzed in detail in the following sections. Additional experiments are shown in Supporting Information: one with the same catalyst, but a lower reaction temperature (525 °C, instead of 550 °C, Figures S6–S8) and another one to check the reproducibility with another batch of the same catalyst under the exact same conditions as used for Figure 3 (Figure S9).

#### Carbon Growth Rate in Time

In order to directly compare the carbon growth rate observed during in situ TEM with the in situ TGA carbon growth rate, we measured the carbon growth rate for particles from the TEM image series. For a full comparison, we required information about: (i) the amount of Ni in a particle, (ii) the carbon fiber length, (iii) the inner and outer fiber diameter, (iv) the time duration of the growth, and (v) the time after the start of the reaction when the growth occurred. Figure S3 illustrates how we extracted all this information from the TEM data, which allowed us to determine the carbon growth rate *r*_C_ in g_C_ g_Ni_^–1^ min^–1^ for individual particles. The growth rate was normalized to the amount of Ni, as this is the active element in the reaction (Cu does not grow CNFs on its own under these conditions^2^). Measured particles in the TEM experiments contained both Ni and Cu in similar ratios with small deviations (average: 70.1 ± 9.8 wt % Ni) as shown in Figure S7. We took a weighted average of ∼10 particles that grew fibers at a similar time after the reaction started. Since larger particles are heavier and contribute more to g_C_ g_Ni_^–1^ min^–1^ in the TGA measurements, the average carbon growth rate ⟨*r*_C_⟩ measured with TEM was weighted by the volume of the NiCu particle *V* for a faithful comparison to the TGA measurements,
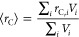
1

**Figure 4:**
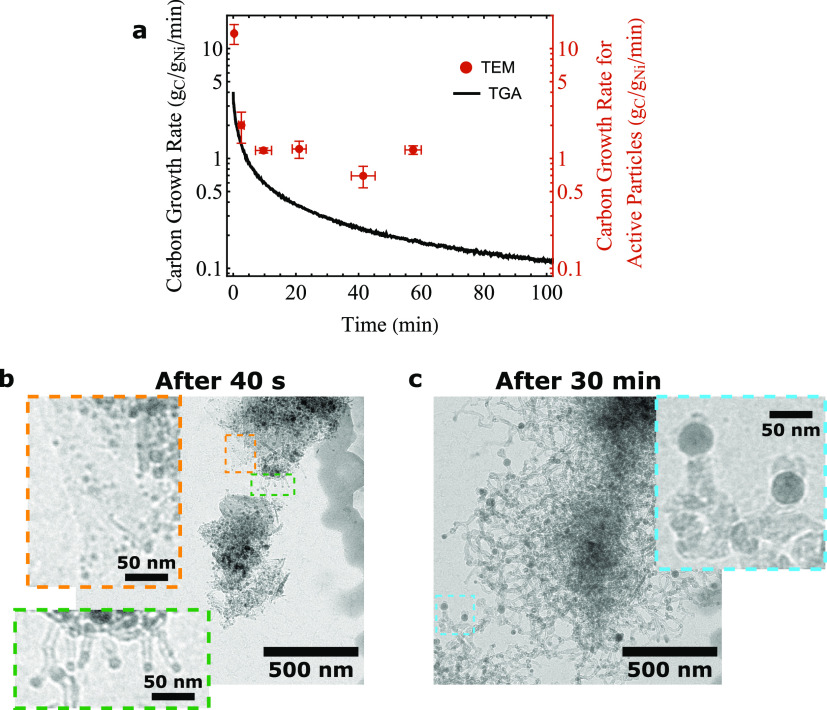
(a) Direct comparison between the carbon growth rate in time measured from in situ TGA and in situ TEM data. The temperature was 550 °C and the feed contained 5% H_2_, 45% CH_4_, and 50% Ar at 1 bar in both experiments. (b) Observation using in situ TEM 40 s after the reaction gas was introduced. Small particles (<8 nm) did not nucleate any fibers (orange dashed box). Medium-sized particles (8–16 nm) particles grew carbon quickly in the first 40 s (green dashed box). (c) In situ TEM observation 30 min after the reaction gas was introduced. Mostly large particles (>16 nm) are still growing fibers (blue dashed box).

The carbon growth rate over time is shown in Figure 4a. The decrease of the carbon growth rate over time shows a similar trend for both measurement techniques. In both the in situ TGA and TEM experiments, the carbon growth rate decreased by an order of magnitude in the first 20 min. While the trends for the carbon growth rate from TGA and TEM are similar, the absolute values do not match. In the following paragraphs, we first discuss the matching trend, and subsequently the difference in the absolute values.

##### Active Particles and Their Deactivation

Let us discuss the result in Figure 4a in more detail, starting with a similar trend in the carbon growth rate measured from both techniques. From in situ TEM observations, we found that relatively small particles (*d* ≈ 8–16 nm) grow fibers quickly in the 1st minute of the reaction (Figure 4b, green box, Movie S2). The majority of the particles that grew carbon quickly deactivated within the first few minutes, which correlates well with the sharp decrease in the TGA carbon growth rate in the first few minutes. The catalyst after 5 min under reaction conditions in the TGA looked similar to the catalyst observed using in situ TEM (Figures 3f,g and S8a). It was furthermore observed that the particles smaller than 16 nm in diameter were encapsulated by carbon (Figure S8c). This means that the metal catalyst particles remain in the product after the reaction, which is not unusual. These particles are difficult to remove, but treatments with dilute nitric acid combined with heat treatments can be used to remove the metal.^50^ Next, we look into the transition from fast, but unstable, carbon growth in the 1st minutes of the reaction to slow, but steady, carbon growth in the latter stages of the reaction in both the in situ TGA and TEM measurements. It was found that in the latter stages of the reaction, the active particles had increased in size compared to the beginning of the reaction (Figure 4c and Movies S3 and S4). This had two reasons. First, as mentioned, highly active smaller particles (*d* ≈ 8–16 nm) were deactivated early in the reaction. Second, active particles coalesced during the reaction. These larger particles grew carbon at a significantly slower rate than the smaller particles did at the start, but remained active for a much longer time. This is the reason that the carbon growth rate did not decrease quickly anymore at longer reaction times in the TGA measurements (Figure 4a). The decrease in the carbon growth rate observed in the TGA experiments is therefore partly due to smaller particles deactivating quickly, but also due to the slower growth rate (compared to the smaller particles) of larger particles that continue growing carbon in the latter stages of the reaction.

It is also notable that the larger particles (*d* > 20 nm) tend to grow carbon from multiple sides of the particles, resulting in what is often called “octopus-like” growth in the literature,^51−53^ and is often found for NiCu particles. However, unlike the name suggests, our in situ study shows that the growth of carbon mostly happens on 4 sides of the particles (50 nm or less in diameter) or less at any given time.

##### Inactive Particles

**Figure 5:**
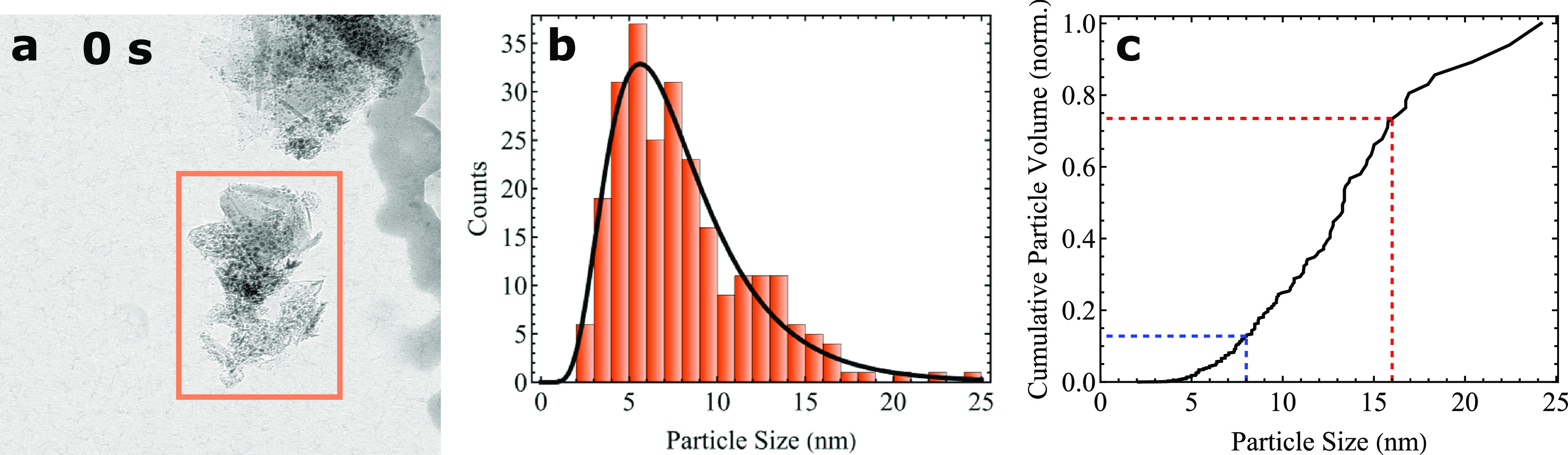
Particle size at the start of the reaction. (a) Area in which the particle sizes were measured at the start of the reaction. (b) Particle size distribution at *t* = 0 s. The volume-averaged particle size was 9.8 ± 4.7 nm. (c) Cumulative volume of particles as a function of particle size. The dashed blue lines indicate that all particles below 8 nm (which tend to not grow CNFs at all) contribute to only 12.5% of the volume of all particles. However, as the red dashed lines indicate, 75% of the volume of particles is in particles below 16 nm, which tend to deactivate within the 1st minute of the reaction.

**Figure 6:**
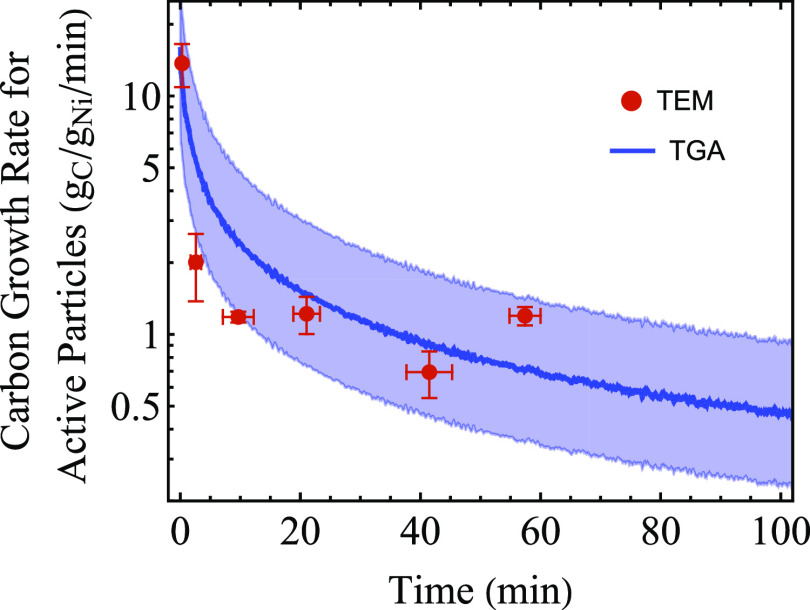
Carbon growth rate for active particles over time for both in situ TEM and TGA experiments. Here, the observation from in situ TEM experiments that only 25% of Ni is in particles that grow CNFs after the initial stages of the reaction are used to correct the TGA curve. The upper and lower boundaries of the TGA curve show the case for which, instead, 12.5 or 50% of Ni was in particles that grow CNFs, respectively, which we estimated as our uncertainty in determining how many particles grew CNFs. The temperature was 550 °C, and the feed contained 5% H_2_, 45% CH_4_, and 50% Ar at 1 bar in both experiments.

Next, we discuss the discrepancy between the absolute values of the carbon growth rate determined from in situ TEM and TGA experiments (Figure 4a). The carbon growth rate measured in TGA is the average over all Ni in the sample, while the growth rate measured by TEM originates only from the active NPs. Importantly, we observed in our in situ TEM experiments that a majority of NPs did not grow any CNFs. Mainly small particles (*d* < 8 nm) did not nucleate fibers at all (Figure 4a, orange box). As mentioned earlier, we also found that particles below 16 nm were deactivated within the first few minutes of the reaction. To explain the difference in the absolute values of the carbon growth rate measured from TGA and TEM, we counted how many particles were larger than 16 nm, as these remained active throughout the reaction. We therefore examined a representative part of the catalyst (Figure 5a, orange box), and measured the sizes of the particles at *t* = 0 s (Figure 5b). Compared to the particle size of the reduced catalyst (Figure 1b), there are larger particles at the start of the reaction. This was due to particles growing in metal-dense areas at high temperatures the instant that the reaction gas was introduced. This is important because the vast majority of the particles measured after reduction (Figure 1b) were smaller than 8 nm, and almost all of them were smaller than 16 nm. Hence, if particle growth had not taken place at the start of the reaction, almost direct deactivation of the catalyst would have occurred. Still there were many small particles at the start of the reaction (Figure 5b, 60% of all particles were smaller than 8 nm), which were shown to not nucleate CNFs. These small particles, due to their volume, consisted of only a fraction of the total amount of Ni in the sample. Figure 5c shows that only ∼12.5% of the total volume of Ni is in particles smaller than 8 nm at the start of the reaction. These smaller particles did not nucleate CNFs, and they were encapsulated with carbon (Figure S4). We estimated that this encapsulation with carbon contributes less than 0.25 g_C_ g_Ni_^–1^ to the total carbon yield, which is negligible in our experiments. As such, these small particles do not contribute meaningfully to the TGA result and, therefore, do not impair the comparison between the TGA and TEM results significantly. However, the particles below 16 nm in size contained ∼75% of all Ni in the catalyst (Figure 5c). As such, after their deactivation during the initial few minutes of the reaction, only 25% of Ni in the sample was contributing to carbon growth. The carbon growth rate measured from in situ TEM in Figure 4a was higher than the TGA growth rate because only the particles that grew CNFs were taken into account. When corrected for 75% of Ni not contributing to the growth (Figure 5c), the carbon growth rates measured via in situ TEM and TGA match remarkably well, as shown in Figure 6. The in situ TEM experiments were therefore validated with in situ TGA measurements for these conditions and could explain the overall evolution of the growth rate observed by understanding the underlying kinetics of the carbon growth on the different groups of individual metal nanoparticles.

## Conclusions

In summary, we measured the carbon growth rate from individual particles using in situ TEM with a suitable electron dose rate as well as the average carbon growth under the same conditions via TGA. This allowed us to understand the evolution of the carbon growth rate over time. First, it turned out that a significant fraction of particles (those with *d* < 8 nm) did not grow CNFs at all. Particles that were slightly larger (*d* ≈ 8–16 nm) grew carbon quickly at the start of the reaction but deactivated quickly. This rapid initial growth caused the high carbon growth rate at the start of the TGA experiment, which decreased quickly due to the deactivation of those particles. The relatively stable but slow carbon growth rate in the latter stages of the reaction was due to larger particles (*d* > 16 nm). After the 1st minutes, 75% of all Ni in the sample was present in inactive particles. There was a good quantitative agreement between the sum of the growth rates of individual metal nanoparticles measured with TEM and the overall growth rate evolution in time measured with in situ TGA.

Our experiments showed that in situ TEM experiments are able to give reliable results on CNF growth for low electron dose rates at relevant pressure conditions. The in situ TEM experiments showed that small particles did not nucleate fibers or deactivate quickly, which suggests that an annealing step before starting the reaction could improve the catalytic reaction by increasing the particle size. All in all, the combination of in situ TEM and TGA measurements is powerful and complementary and allows us to understand how the effective growth rate depends on the properties of individual catalyst nanoparticles such as particle size, particle composition, and interaction with the support material.

## Supporting Information Available

The following files are available free of charge. The Supporting Information is available free of charge at https://pubs.acs.org/doi/10.1021/acs.jpcc.3c02657.

Details on the influence of the electron beam, method, and grown carbon structures and description of additional experiments at 525 and at 550 °C with another batch (PDF)
Carbon nanofiber growth from carbon-supported NiCu nanoparticles at 0.1 and 52 e^–^ Å^–2^ s^–1^ (Movie S1) (MP4)
Carbon nanofiber growth at 550 °C with a feed of 5% H_2_, 45% CH_4_, and 50% Ar at 1 bar (Movie S2) (MP4)
Carbon nanofiber growth at 550 °C with a feed of 5% H_2_, 45% CH_4_, and 50% Ar at 1 bar (MP4)
Carbon nanofiber growth at 550 °C with a feed of 5% H_2_, 45% CH_4_, and 50% Ar at 1 bar (Movie S4) (MP4)
