# Supplementary material for: Carbon Nanofiber Growth Rates on NiCu Catalysts: Quantitative Coupling of Macroscopic and Nanoscale In Situ Studies
Source: J Phys Chem C Nanomater Interfaces. 2023 Aug 4;127(32):15766–74. doi: 10.1021/acs.jpcc.3c02657 (PMC10440819; doi:10.1021/acs.jpcc.3c02657)
Supplement: Supplementary file 1 — jp3c02657_si_001.pdf [file jp3c02657_si_001.pdf]

Supporting information for “Carbon Nanofiber Growth Rates on NiCu Catalysts: Quantitative Coupling of Macroscopic and Nanoscale In-Situ Studies”

Tom A.J. Welling[a†], Suzan E. Schoemaker[a†], Krijn P. de Jong[a], and Petra E. de Jongh[a]

[a]Materials Chemistry & Catalysis, Debye Institute for Nanomaterials Science, Utrecht University, Universiteitsweg 99, 3584 CG Utrecht, The Netherlands
[†]These authors contributed equally.

Contents

1 Influence of the electron beam **S2**
1.1 Figure S1: Imaging with and without hydrogen . . . S2
1.2 Figure S2: Electron dose rate effects . . . S3

2 Method **S4**
2.1 Figure S3: Method of measuring carbon growth rate . . . S4
2.2 Discussion on challenges and caveats . . . S5

3 Carbon structures **S6**
3.1 Figure S4: Carbon encapsulation . . . S6
3.2 Figure S5: Octopus-like growth . . . S7

4 Experiments at 525 °C **S8**
4.1 Figure S6: Carbon growth rate in time . . . S8
4.2 Figure S7: NiCu composition . . . S9
4.3 Figure S8: Images after 5 minutes in TGA . . . S10

5 Reproducibility with another batch **S11**
5.1 Figure S9: The same two catalysts under the same conditions . . . S11

6 Full carbon growth rate data from TGA measurement **S12**
6.1 Figure S10: Full carbon growth rate data from TGA measurement . . . S12

7 Supporting Movie captions **S13**

1 Influence of the electron beam

1.1 Figure S1: Imaging with and without hydrogen

Figure S1 shows images of the catalyst after being at reaction conditions for at least an hour. In the areas that were not imaged, carbon fiber growth occurred for all conditions tested. However, the areas that were imaged only showed carbon fibers when hydrogen was present in the feed. As such, hydrogen prevents an inhibition of growth due to the electron beam.

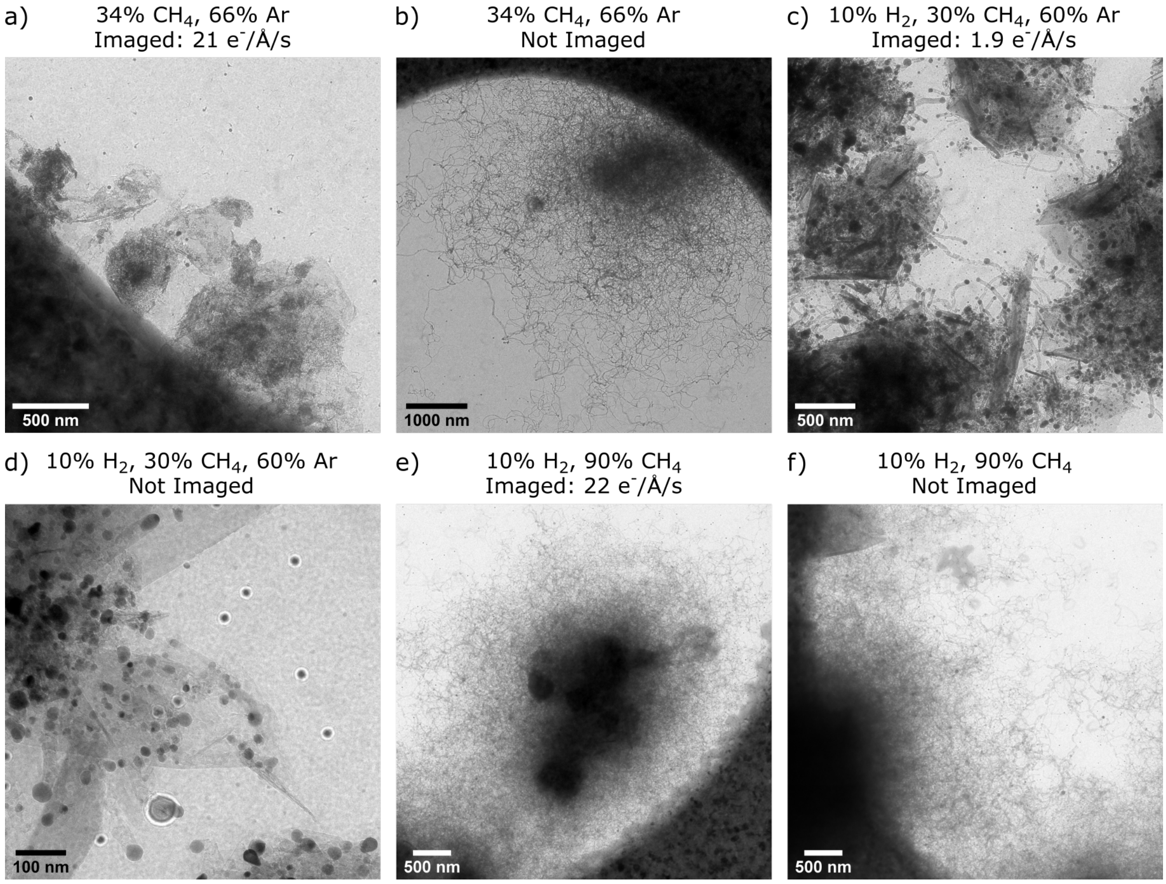

Figure S1: Carbon nanofiber growth observed in a gas cell at spots that were continuously imaged (a, c, and e) or only imaged after the reaction (b, d, and f) at a constant temperature of 600 °C. The images were taken after the sample had been under reaction conditions for at least an hour. In the areas that were imaged without hydrogen present in the feed, no carbon fiber growth occured.

1.2 Figure S2: Electron dose rate effects

Figure S2a shows the carbon structure length growth rate as function of electron dose rate used to image the sample. This data was obtained by measuring the fiber length that was grown over several minutes for more than 10 particles per electron dose rate condition during an experiment. In Figure S2b-c it is shown that both the time of the measurement after the start of the reaction and the average particle size had no influence on the carbon structure length growth rate, compared to the electron dose rate (as shown in Figure S2a).

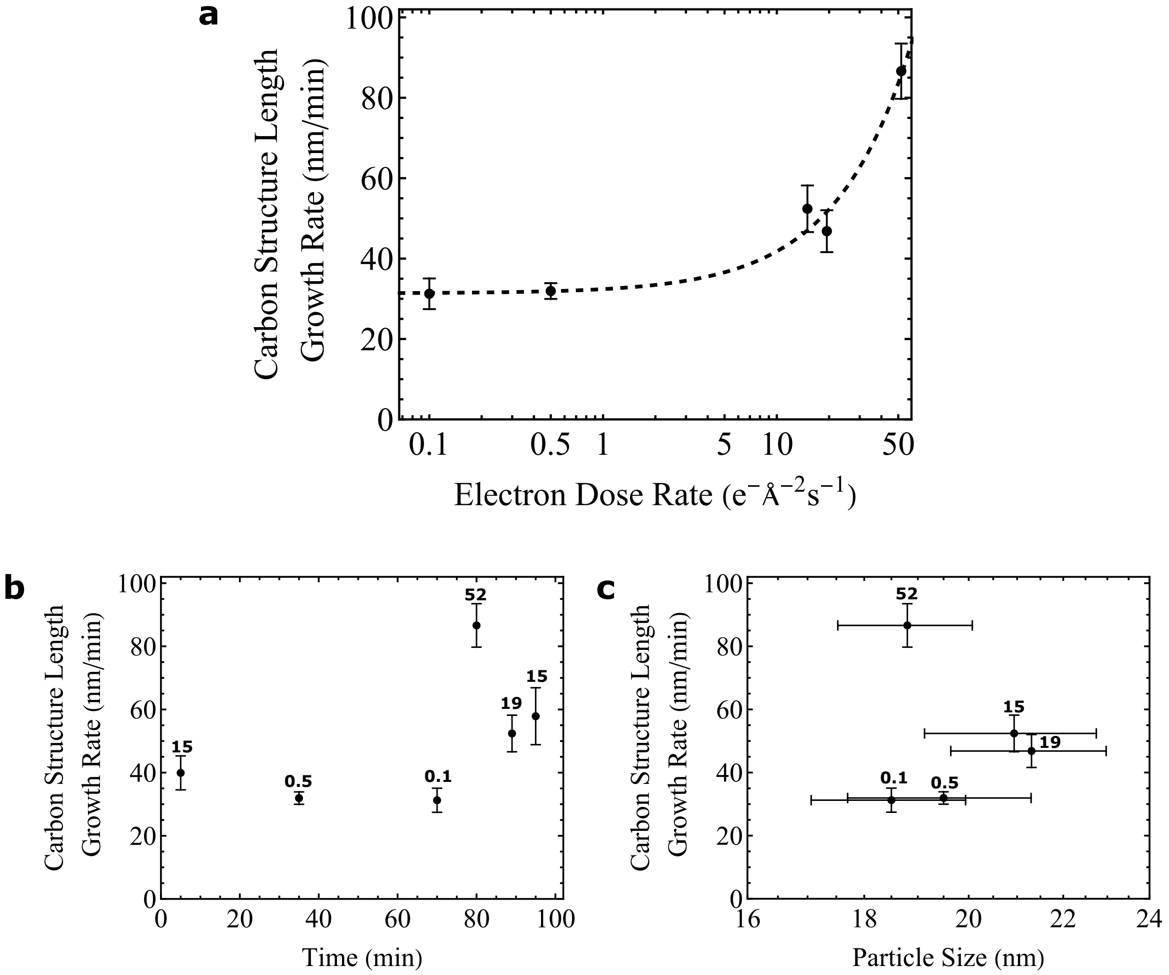

Figure S2: a) Average carbon nanofiber length growth rate of active NiCu particles versus the electron dose rates measured from in-situ TEM image series. The dashed line is a linear fit through the data (Note: the horizontal axis is logarithmic). b-c) Average carbon nanofiber length growth rate for different electron dose rates plotted versus time since the start of the reaction (b) and average particle size growing the measured fibers (c). The temperature was 550 °C and the feed contained 5% H_2, 45% CH_4, 50% Ar at 1 bar.

2 Method

2.1 Figure S3: Method of measuring carbon growth rate

Figure S3 shows how we obtain the data required to calculate the carbon growth rate in $g_C g_{Ni}^{-1} min^{-1}$ as discussed in the main text. For a full comparison, we required information about: (i) the amount of Ni in a particle, (ii) the carbon fiber length, (iii) the inner and outer fiber diameter, (iv) the time duration of the growth, and (v) the time after the start of the reaction when the growth occured. Figure S3 illustrates how we extracted all this information from the TEM data, which allowed us to determine the carbon growth rate r_C in $g_C g_{Ni}^{-1} min^{-1}$ for individual particles.

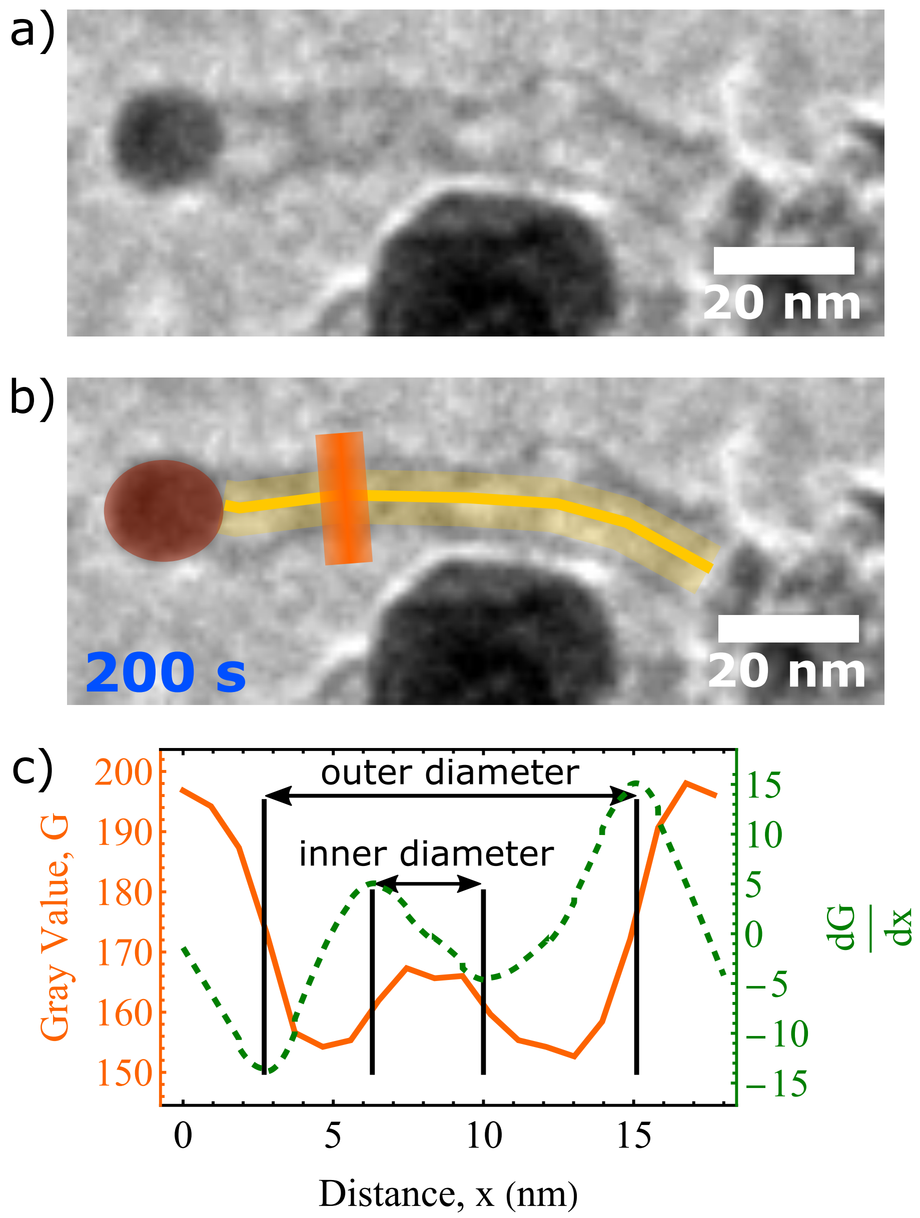

Figure S3: Method used to obtain the carbon growth rate of individual particles from in-situ TEM data in $g_C g_{Ni}^{-1} min^{-1}$. a) Original image. b) Image containing the growth time (blue), particle size (red), fiber length (yellow), and fiber cross section (orange). c) Determination of the inner and outer diameter of the fiber from the cross section in b.

2.2 Discussion on challenges and caveats

However, there are some challenges in determining the carbon growth rate from TEM observations. One concern is the resolution and pixel size, which may impact the accuracy of the determination of the inner and outer diameter of the fiber, especially for thinner fibers. However, since both our pixel size and resolution are slightly below 1 nm, while the fibers are typically between 10 and 30 nm, no large influence is expected. Second, we assumed that the 2D projection of the fibers gave a good indication of their diameter and that the fibers were cylindrical and not anisotropic. Additionally, we could not measure fibers that grew in the z-direction, parallel to the electron beam path, as they move in and out of focus conditions. However, in practice we observed that the vast majority of the fibers grew more or less parallel to the gas-cell window, which hints towards an interaction with the SiN surface of the chips. Another difference with large-scale reactors is that we observed only a small fraction of the catalyst, and preferably in a less dense catalyst domain, while in the reactor also many denser structures are present. The space for CNFs to grow into is therefore somewhat different between reactors and in-situ TEM experiments. Last, the flow rate of gas through the nanoreactor is high with a gas hourly space velocity (GHSV) of approximately $10^{10}\,h^{-1}$. This is about an order of magnitude higher than for the TGA experiments. It is therefore unlikely to impair the comparison between the TEM and TGA results. Overall, while there are some differences between experiments on a large scale in a reactor and our experiments in a nanoreactor, we believe our nanoscale experiments are still representative for what happens in larger reactors.

3 Carbon structures

3.1 Figure S4: Carbon encapsulation

Figure S4 shows a small particle encapsulated by carbon after being under reaction conditions. A simple calculation clarifies that this particular particle, with a diameter of 6 nm and a carbon shell of 3 nm thickness, leads to a contribution of 2.35 $g_C g_{Ni}^{-1} min^{-1}$ by its deactivation. Based on more TEM images of deactivated particles, the average contribution of particles encapsulated by carbon is 1.67 ± 0.34 $g_C g_{Ni}^{-1} min^{-1}$ per particle. Only approximately 12.5% of Ni is in particles that deactivate like this (main text). As such, the total contribution to the TGA measurements of this type of deactivation is less than 0.25 $g_C g_{Ni}^{-1}$.

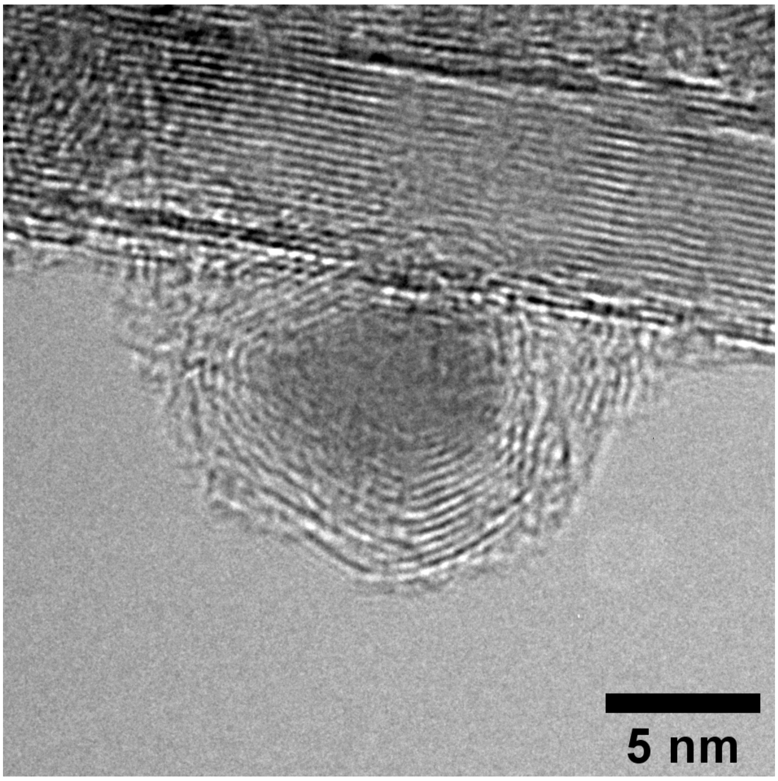

Figure S4: Encapsulation of a small particles after the reaction in TGA. The temperature was 550 °C and the pressure was 1 bar with a gas composition of 5% H_2, 45% CH_4, 50% Ar.

3.2 Figure S5: Octopus-like growth

Figure S5a shows an octopus-like structure grown from a larger NiCu particle. From panel b we observe that the graphite layers are stacked parallel to the parallel surface.

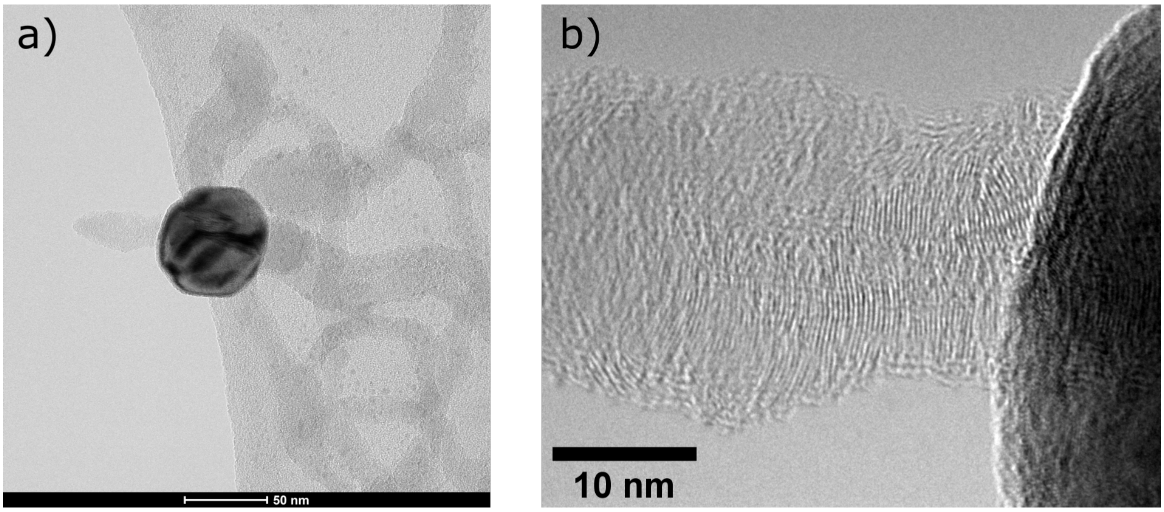

Figure S5: a) Various fibers grown from a large particles after the reaction in TGA. The temperature was 550 °C and the pressure was 1 bar with a gas composition of 5% H_2, 45% CH_4, 50% Ar. b) Zoom-in image of the structure in a, showing the graphene is mostly arranged in a coinstack configuration.

4 Experiments at 525 °C

4.1 Figure S6: Carbon growth rate in time

In order to test if our conclusions of the main text hold for slightly different conditions, we also did experiments at 525 °C, instead of 550 °C. Figure S6a shows again a similar trend between the carbon growth rate obtained from in-situ TEM and TGA measurements. The growth rate was slightly lower than for 550 °C, as expected. Panels b-d indicate similar trends as for 550 °C, where small particles grew carbon quickly at the start of the reaction, while larger particles were mainly growing carbon later on in the reaction.

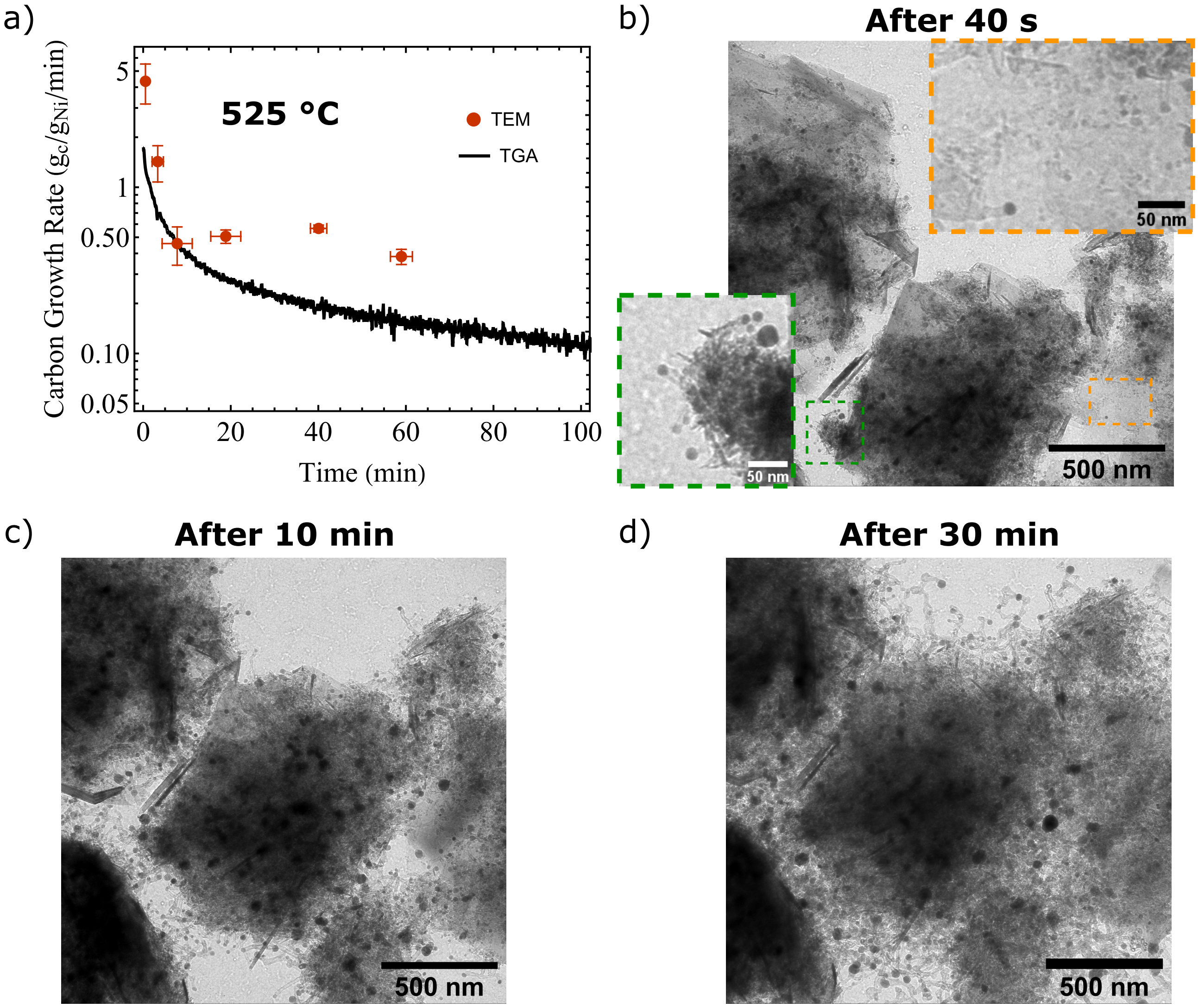

Figure S6: Comparison between in-situ TEM and TGA measurements at a reaction temperature of 525 °C. The pressure was 1 bar with a gas composition of 5% H_2, 45% CH_4, 50% Ar. a) Carbon growth rate measured from in-situ TEM and TGA. For the in-situ TEM measurement, only active particles were measured. b-d) TEM images showing an overview of the catalyst 40 s, 10 minutes, and 30 minutes after the start of the reaction.

4.2 Figure S7: NiCu composition

Figure S7a shows an EDX map of the elements Ni and Cu after an in-situ TEM experiment. Since we could identify the particles within the movies made during the experiment, we could relate the growth rate to the elemental composition of the particles. Panel b shows there is no clear correlation between the particle size and the composition. There was also no clear trend observed between the composition and the carbon growth (panel c). Panel d shows that the correlation between particle size and carbon growth was much more pronounced.

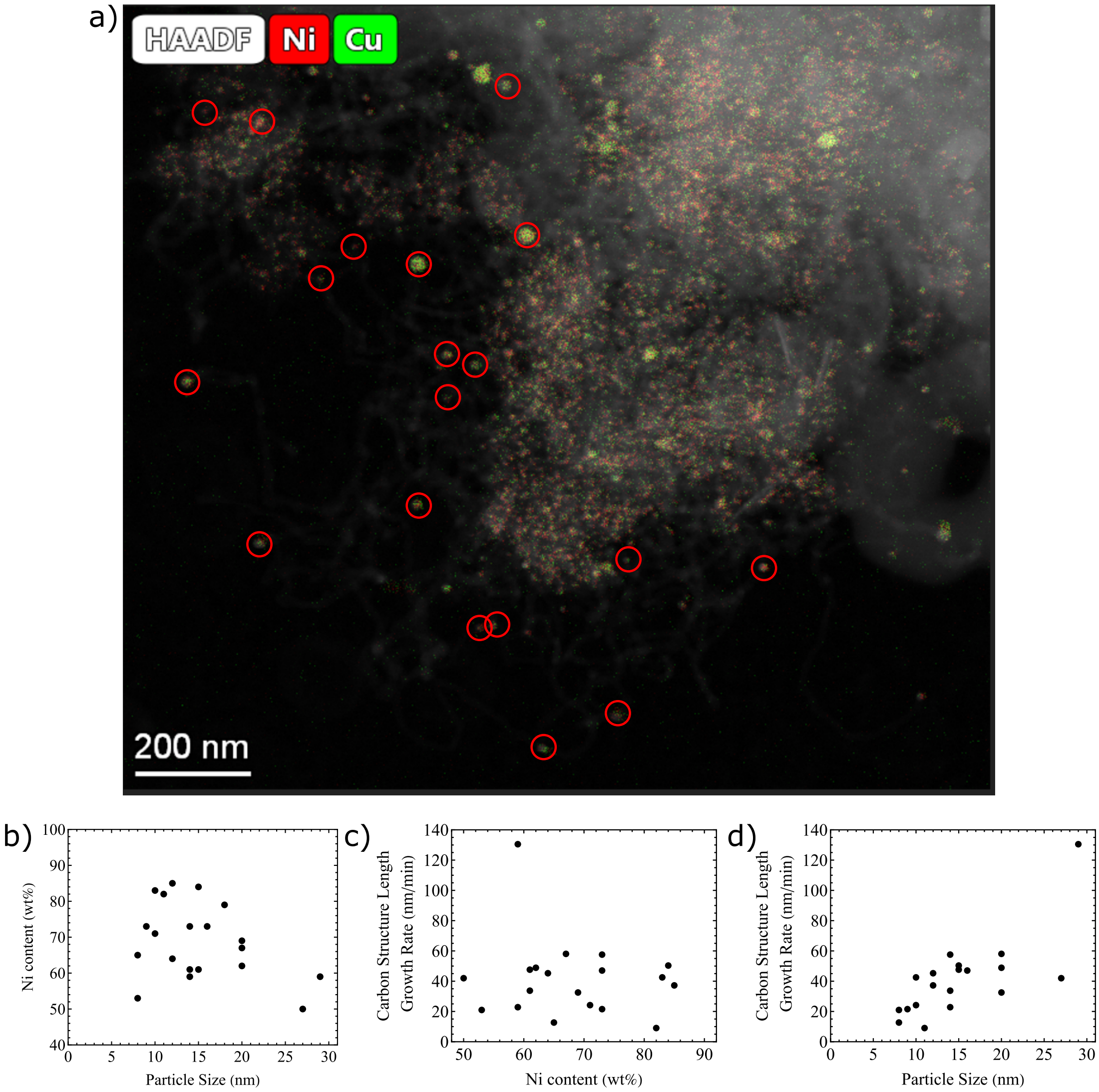

Figure S7: a) EDX map of the catalyst after an in-situ TEM experiment. The red circles indicate particles that grew CNFs for which the growth length could be measured from the in-situ experiment. b) Ni content versus particle size, c) carbon growth rate vs Ni content, and d) carbon growth rate vs particle size for the particles indicated in a. The temperature was 525 °C and the pressure was 1 bar with a gas composition of 5% H_2, 45% CH_4, 50% Ar.

4.3 Figure S8: Images after 5 minutes in TGA

To check whether the in-situ TEM experiments represent the in-situ TGA experiments, we stopped a TGA experiment after 5 minutes and observed the result in conventional TEM. Similar to the in-situ TEM experiments (Figure 4 in the main text), smaller particles were moved outside the support area due to carbon growth after 5 minutes (Figure S8).

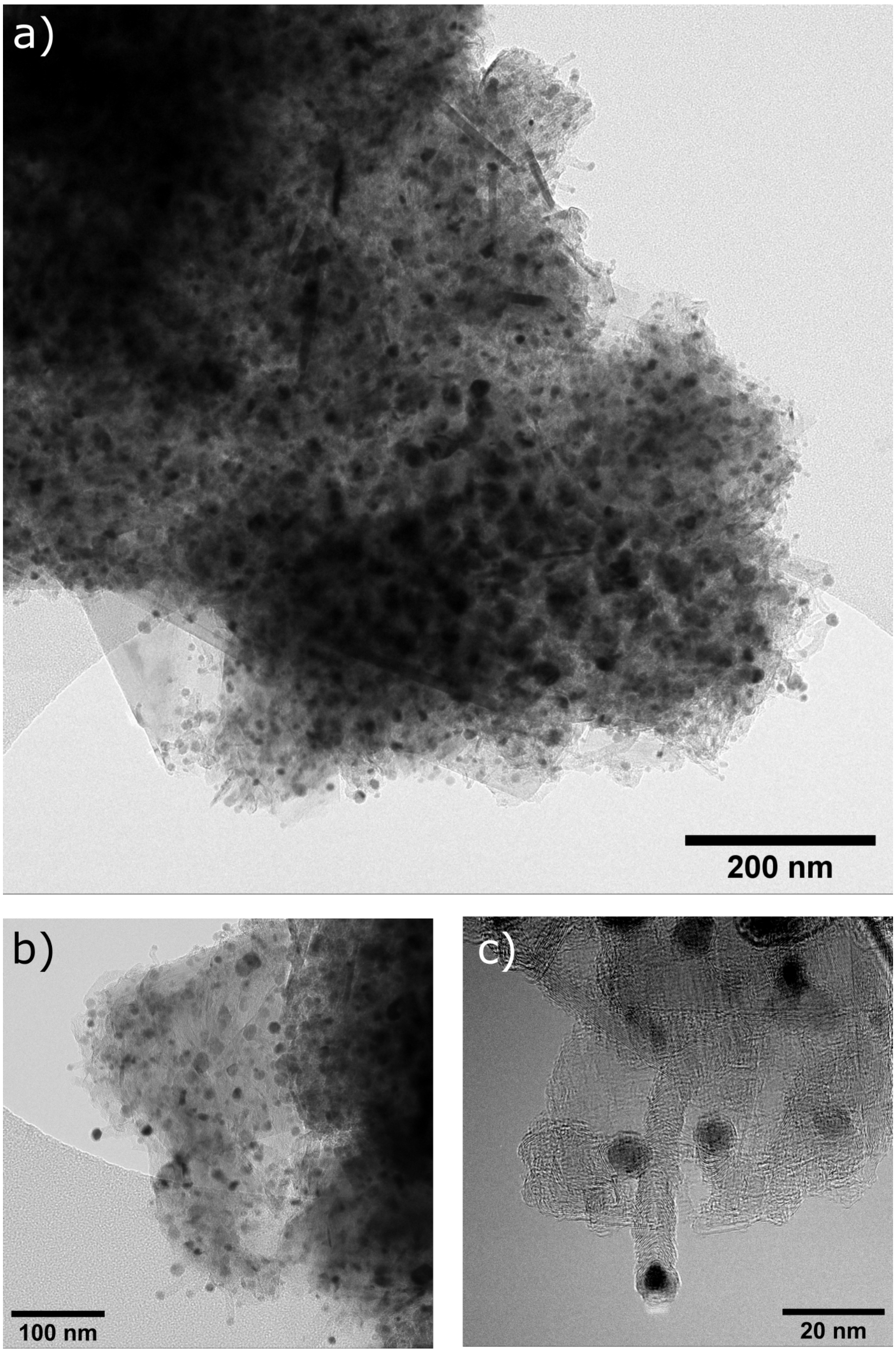

Figure S8: Catalyst after 5 minutes under reaction conditions in TGA. The reaction temperature was 525 °C. The pressure was 1 bar with a gas composition of 5% H_2, 45% CH_4, 50% Ar. a-b) Typical overviews. c) High-resolution image showing fishbone-structured carbon and encapsulation of deactivated particles.

5 Reproducibility with another batch

5.1 Figure S9: The same two catalysts under the same conditions

Two batches of the same catalyst were subjected to the same conditions in an in-situ TEM experiments to check reproducibility. Figure S9 shows that the carbon growth rate for active particles in time was relatively similar for both samples.

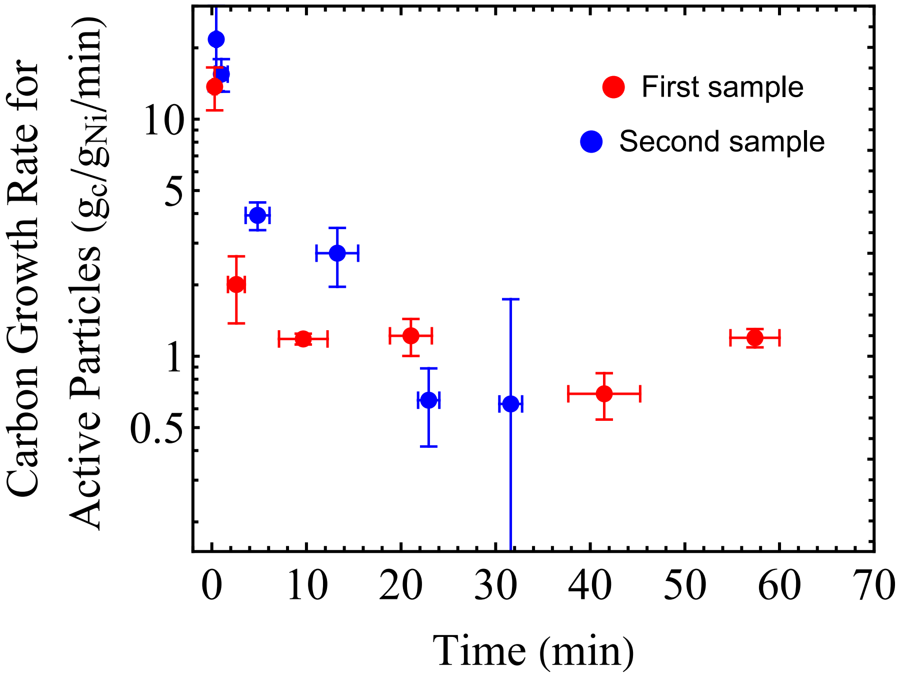

Figure S9: Carbon growth rate measured from in-situ TEM as function of time for two different 11 wt% Ni/4 wt% Cu catalyst samples synthesized the same way. Both reactions were performed under the same conditions. The temperature was 550 °C. The pressure was 1 bar with a gas composition of 5% H_2, 45% CH_4, 50% Ar

6 Full carbon growth rate data from TGA measurement

6.1 Figure S10: Full carbon growth rate data from TGA measurement

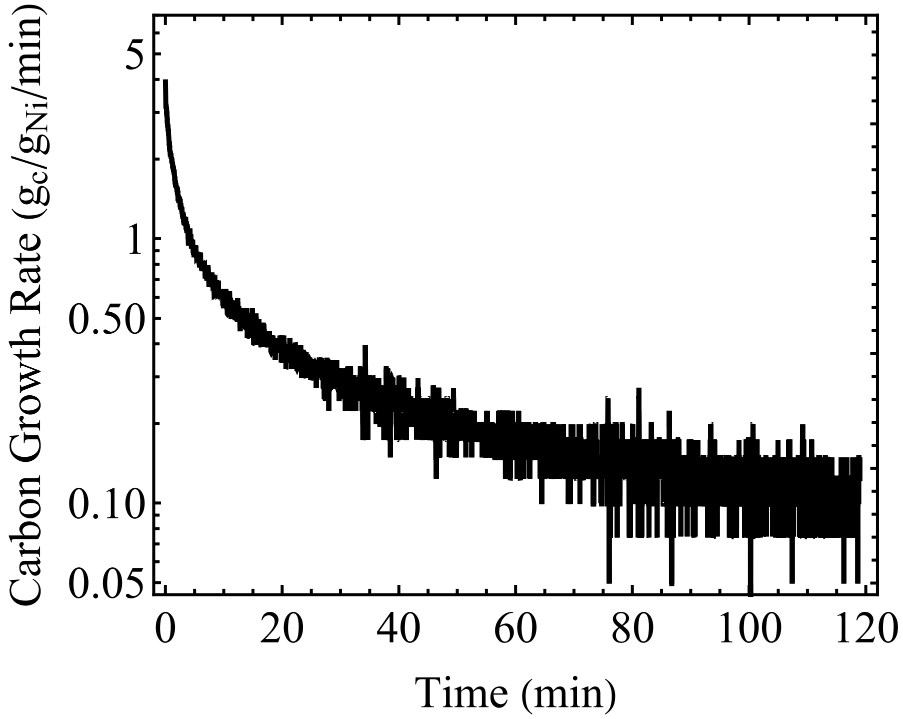

Figure S10: Full, unsmoothed carbon growth rate measured with TGA as shown in Figure 2 in the main text. The temperature was 550 °C. The pressure was 1 bar with a gas composition of 5% H_2, 45% CH_4, 50% Ar

7 Supporting Movie captions

- Supporting Movie S1: Movies of carbon nanofiber growth from carbon-supported NiCu nanoparticles at 0.1 and $52\,e^{-}\,Å^{-2}\,s^{-1}$. The pressure was 1 bar with a gas composition of 5% H_2, 45% CH_4, 50% Ar and the temperature was 550 °C.
- Supporting Movie S2: Movie of carbon nanofiber growth at 550 °C with a feed of 5% H_2, 45% CH_4, 50% Ar at 1 bar. The electron dose rate was $7.7\,e^{-}\,Å^{-2}\,s^{-1}$. The reaction gas was introduced at $t = 0\,s$. Part 1: 0-350 s.
- Supporting Movie S3: Movie of carbon nanofiber growth at 550 °C with a feed of 5% H_2, 45% CH_4, 50% Ar at 1 bar. The electron dose rate was $7.7\,e^{-}\,Å^{-2}\,s^{-1}$. The reaction gas was introduced at $t = 0\,s$. Part 2: 352-710 s.
- Supporting Movie S4: Movie of carbon nanofiber growth at 550 °C with a feed of 5% H_2, 45% CH_4, 50% Ar at 1 bar. The electron dose rate was $7.7\,e^{-}\,Å^{-2}\,s^{-1}$. The reaction gas was introduced at $t = 0\,s$. Part 3: 1020-1380 s.
